# Cyclic stretch induced-retinal pigment epithelial cell apoptosis and cytokine changes

**DOI:** 10.1186/s12886-017-0606-0

**Published:** 2017-11-22

**Authors:** Shen Wu, Qingjun Lu, Ningli Wang, Jingxue Zhang, Qian Liu, Meng Gao, Jinqiu Chen, Wu Liu, Liang Xu

**Affiliations:** 1 Beijing Institute of Ophthalmology; Beijing Tongren Eye Center; Beijing Tongren Hospital, Capital Medical University; Beijing Ophthalmology & Visual Sciences Key Laboratory, Beijing, 100005 China; 2 Beijing Tongren Eye Center; Beijing Tongren Hospital, Capital Medical University; Beijing Ophthalmology & Visual Sciences Key Laboratory, Beijing, 100005 China; 3 China-Japan Friendship Hospital, Beijing, 100029 China

**Keywords:** Retinal pigment epithelium, Apoptosis, Cytoskeleton, Age-related macular degeneration, Mechanical stretch

## Abstract

**Background:**

The pathogenesis of age-related macular degeneration (AMD) is complex. It has been shown that vitreomacular traction (VMT) plays a role in the pathogenesis of AMD. We speculate that the continuous stretch induced by VMT might impair the function of retinal pigment epithelium (RPE) cells and it might also be involved in the progression of AMD.

**Methods:**

Cultured ARPE-19 cells were subjected to cyclic stretch on the Flexcell Strain system at a level of 25% increment on the surface area for 8 h, 14 h, 20 h, 24 h. In another group, the stretch was withdrawn at 14 h and the cell cultured for another 6 h. Then, we observed the changes in morphology, apoptosis and expression of interleukin 6 (IL6) and vascular endothelial growth factor (VEGF) in RPE cells under stretch.

**Results:**

We found that stretch induced the RPE cells to change from a spreading shape into a rounded shape, and that the morphological changes were positively correlated with the duration of the stretch. The expression of pFAK397 and pRac1/cdc42 were elevated in a time-dependent fashion. The stretch resulted in an increase in the apoptosis ratio, with Bcl2, Bax and p53 also showing time-dependent changes. In addition, up-regulation of IL6 and VEGF expression levels was also observed. After withdrawal of the stretch, all of these changes were significantly diminished.

**Conclusion:**

Stretch may induce morphological, cell apoptosis, and up-regulation of cytokines changes in RPE cells, indicating that cyclic stretching may participate in the progression of AMD by impeding the functions of the RPE.

## Background

Age-related macular degeneration (AMD) is a leading cause of blindness in elderly people throughout the world [1]. Several hypotheses have been proposed concerning the pathogenesis of AMD, suggesting that oxidative stress [2, 3], vascular pattern, immunity, inflammation [4] and heredity [5, 6] may play a role; however, there is no comprehensive and universally-accepted explanation to fully clarify the pathogenesis of AMD, which reflects its underlying complexity and diversity.

In recent years, a growing body of clinical evidence has shown that a pathological change in vitreomacular adhesion (VMA) is present in most patients with AMD [7–10]. This VMA change means that the vitreous cortex may exert continuous traction on the macular retina; the higher the severity of VMA, the more rapid the progression of AMD [11]. On the other hand, resolution of VMA using ocriplasmin [7] or vitrectomy is likely to delay the progression of AMD [12]. We speculate that the continuous stretch induced by VMA may impair retinal cells, particularly the superficial retinal pigment epithelium (RPE) cells, and thereby participate in the development and progression of AMD.

It has been shown that multiple risk factors for AMD are associated with changes in the physiological functions of RPE cells; for example, oxidants can induce apoptosis of RPE cells [13] and abnormal secretion of inflammatory factors [14]. Cigarette smoke extract can also induce apoptosis of RPE cells [15] and up-regulation of vascular endothelial growth factor (VEGF) expression [16], while a high-fat diet may result in up-regulation of interleukin-8 (IL-8) and VEGF expressions in RPE cells [17]. A critical question that remains to be answered is whether the mechanical stretch induced by VMA would lead to AMD-related pathological changes in RPE cells. Prior studies reported that physiological stretch (<20% elongation) might induce changes in the actin filament arrangement of cultured RPE cells in vitro [18, 19] and may also activate small conductance calcium-activated potassium channels to protect RPE cells [20]. Such findings reveal that stretch is able to produce changes in the physiological functions of RPE cells; however, there is no reliable data concerning the effect of long-term pathological stretch on RPE cells, or what will happen after withdrawal of the stretch. Considering this gap in knowledge, it is crucial to investigate the physiological and pathological changes in RPE cells under mechanical stretch, as well as the underlying mechanisms, to provide important insights into the pathogenesis of AMD.

In this study, cyclic stretch was imposed on RPE cells cultured in vitro, resulting in morphological changes in RPE cells from a spreading shape into a rounded shape, as well as a gradual increase in the apoptosis ratio. Additionally, up-regulation of IL6 and VEGF expression levels were observed. After withdrawal of the stretch, all of these alterations were somewhat reversed. As these observed changes in RPE cells are generally consistent with those observed in the pathological process of AMD, we speculate that the stretching intervention may contribute to the pathogenesis and progression of AMD by inducing cytoskeletal pathway abnormalities in RPE cells, mediating cell apoptosis and up-regulating cytokine secretion.

## Methods

### ARPE19 cell culture and mechanical stretch

ARPE-19 cells were purchased from ATCC Cop. and cultured on a 10 cm dish (NEST) in DMEM/F-12 (1:1) medium (Hyclone) containing 10% FBS (Gibco), 2 mM glutamine (Gibco), 100 U/mL penicillin (Gibco), and 100 μg/mL streptomycin (Gibco). The medium was changed every 2 days. Confluent monolayers of cells were trypsinised and passaged every 4–5 d.

ARPE-19 cells (50,000 cells/well) were seeded on BioFlex culture plates coated with Collagen type I (Flexcell International Corp., USA). The low serum medium (DMEM/F-12 with 1% FBS) was changed 24 h before the initiation of the experiment. The cells were subjected to cyclic strain on the Flexcell Strain system (model FX-5000) at a level of distension sufficient to promote an increment of approximately 25% in surface area at the point of maximum distension on the culture surface. Cyclic stretch was performed for 8 h, 14 h, 20 h, and 24 h at 0.125 Hz, without interruptions. In another group, the stretch was withdrawn at 14 h and the cell cultured for another 6 h. All ARPE cells from the two groups were used for subsequent experiments. At least three samples of each condition were examined.

### Fluorescence staining of F-actin

Cells were washed with phosphate buffered saline (PBS) and fixed in 4% formaldehyde solution in PBS for 1 h at room temperature (RT). After three washes with PBS, cells were permeabilized in 0.3% TritonX-100 in PBS for 10 min and then treated with blocking solution (10% goat serum in PBS) for 1 h at RT. Cells were then stained with a 50 mg/rhodamine-labeled phalloid in conjugate solution in PBS for 40 min at RT in the dark and washed three times with PBS to remove unbound phalloid in conjugate. Cells were completely covered with 300 nM DAPI staining solution and incubated for 3 min. Cells were then rinsed several times with PBS. Samples were examined with a ZEISS ObserverZ1 fluorescence microscope equipped with a CCD camera (ZEISS, Germany).

### Western blotting

Proteins were extracted from the stretched ARPE cells and total concentration measured using a BCA protein assay kit (CWBIO, CHINA) according to the manufacturer’s instructions. Equal quantities of proteins per gel lane were separated on 10% polyacrylamide gels by SDS-PAGE and transferred to polyvinylidene fluoride membranes using an electroblotting apparatus (Bio-Rad, USA). Membranes were blocked in 5% non-fat milk/TBS-Tween20 solution, followed by separate incubation at 4 °C overnight with the primary antibodies, specifically GAPDH (1:1000, SantaCruz, No. sc-25,778), integrin β3 (1:1000, abcam, No. ab34409), p-FAK397 (1:1000, CST, No. 3283), cdc42/rac1 (1:1000, CST, No. 4651), p-cdc42/rac1 (1:1000, CST, No. 2461S), Bcl2 (1:1000, CST, No. 2872), Bax (1:1000, CST, No. 2774), p53 (1:1000, CST, No. 2527), IL6 (1:1000, abcam, No. ab6672), and VEGF (1:1000, abcam, No. ab1316). The membranes were then incubated for 1 h at RT with horseradish-peroxidase (HRP)-conjugated secondary antibody (goat anti-mouse/rabbit IgG antibody (1:1000, invitrogen, No. G21240/G21234), diluted to 1:1000 in 0.5% non-fat milk/TBS-Tween20. Membranes were washed 3 times (10 min/wash) with 0.1% TBS-Tween20 after each antibody application. Immuno-labelled proteins were detected using the ECL Plus Detection System (Invitrogen, USA), according to the manufacturer’s instructions. Protein band intensities were quantified using ImagePro analysis.

### Apoptosis assay

Apoptosis was measured with the Annexin V-FITC apoptosis detection kit I (BD Pharmingen), according to the manufacturer’s instructions. Briefly, cells were washed twice with cold PBS and then re-suspended in 500 μl of binding buffer at a concentration of 1 × 10^6^ cells per ml. 5 μl of Annexin V-FITC solution, with 5 μl of PI (propidium iodide, 1 mg/ml) added to these cells at 37 °C for 30 min. The cells were analyzed by flow cytometry within 1 h. Apoptotic cells were counted and represented as a percentage of the total cell count.

### Statistical analysis

All data were presented as mean ± SEM. Quantification was based on data collected from three separate experiments. One-way ANOVA and Tukey multiple pair-wise comparisons were used to determine significant statistical differences, which was considered when *P* < 0.05.

## Results

### Morphological changes of RPE cells

**Fig. 1:**
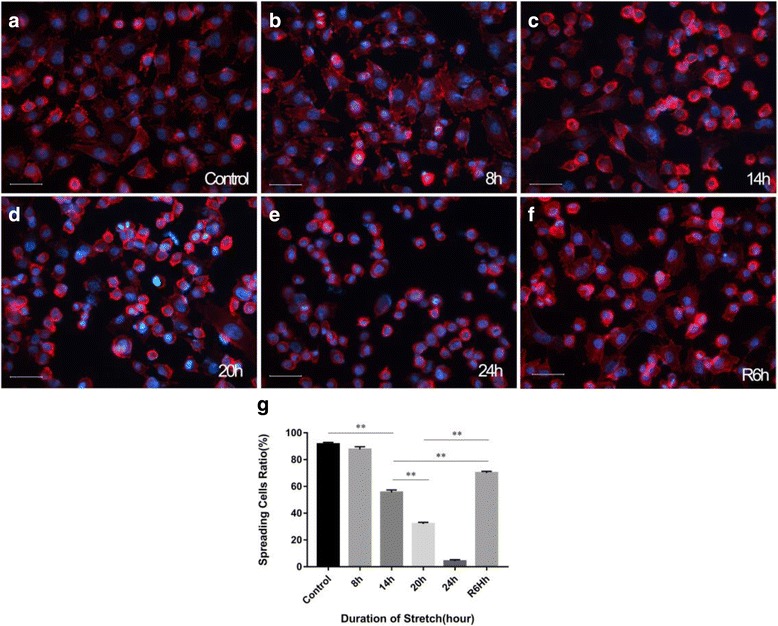
Morphological changes induced by cyclic mechanical stretch. **a**-**f** Phalloidin staining of RPE cells at different time points; **g** The spreading cell ratio at different time points (red fluorescence: phalloidin staining; and blue fluorescence: DAPI nuclear staining). The morphological change of RPE cells was dependent on the duration of the stretch. At 14 h of cyclic stretch, the spreading cell ratio started to decrease sharply (*p* < 0.01). At 6 h after withdrawal of the stretch (R6h), the spreading cell ratio was significantly higher than at 14 h and 20 h (*p* < 0.01)

Phalloidin staining of the actin cytoskeleton was performed to visualise the morphological changes in RPE cells. The RPE cells cultured in vitro exhibited a polygonal shape and were randomly oriented (Fig. 1a). Under cyclic stretch, these cells underwent morphological changes, from a spreading shape to a rounded shape. The extent of change was positively correlated with the duration of the stretch (Fig. 1b-e). At 14 h of cyclic stretch (14 h), the spreading cell ratio started to decrease sharply (*p* < 0.01), was approximately 30.0% at 20 h (Fig. 1d, g; Table 1) and then as low as 4.22% at 24 h (Fig. 1e, g, Table 1). At 6 h after withdrawal of the stretch, the spreading cell ratio returned to about 70% (Fig. 1f, g, Table 1), being significantly higher than at 14 h and 20 h (*p* < 0.01).

**Table 1:** Stretch-induced changes in spreading cell ratio

Duration of the stretch (hour)	Spreading cell ratio (%)
Control	91.58 ± 1.29
8 h	87.63 ± 2.02
14 h	55.56 ± 1.78
20 h	31.94 ± 1.27
24 h	4.22 ± 1.06
R6h	70.07 ± 1.19

### Changes in cytoskeletal regulatory proteins

**Fig. 2:**
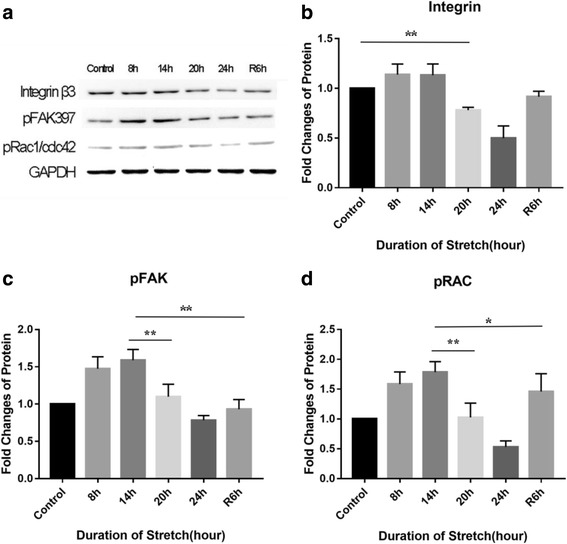
Western blotting analysis of cytoskeleton regulatory protein expression. **a** Coloured bands of integrin signalling pathway-related proteins. **b**-**d** Expression levels of integrin, phosphorylated FAK397 (pFAK397), and phosphorylated Rac1/cdc42 (pRac/cdc42). The integrin expression (**b**) remained almost unchanged during the first 14 h of stretch, but started to decrease significantly at 20 h (*p* < 0.01). The expression of both pFAK397 (**c**) and pRac/cdc42 (**d**) was significantly up-regulated in the first 14 h of stretch (*p* < 0.05), but began to decrease at 20 h (*p* < 0.01)

The morphological changes were regulated by the integrin-FAK-Rac/cdc42 signal pathway. The related proteins were assessed by Western blotting. The expression of integrin β3 remained almost unchanged during the first 14 h of stretch, but started to decrease significantly at 20 h (*p* < 0.01). In the stretch-withdrawn group, no decreasing trend was observed compared to 14 h (Fig. 2b). Up-regulation of the phosphorylation level of FAK397 was noted at 8 h (*p* < 0.05), which was maintained until 14 h; however, a significant down-regulation was seen at 20 h compared to 14 h (*p* < 0.01). In the stretch-withdrawn group, the phosphorylation level of FAK397 was significant lower than at 14 h, and had no differences compared to the control (Fig. 2c). A similar trend (an initial increase followed by decrease) was observed in the phosphorylation level of Rac/cdc42; in the stretch-withdrawn group, Rac/cdc42 and its phosphorylation level tended to decrease after stretch withdrawal at 14 h and almost returned to the control level at R6h (Fig. 2d).

### RPE cell apoptosis

**Fig. 3:**
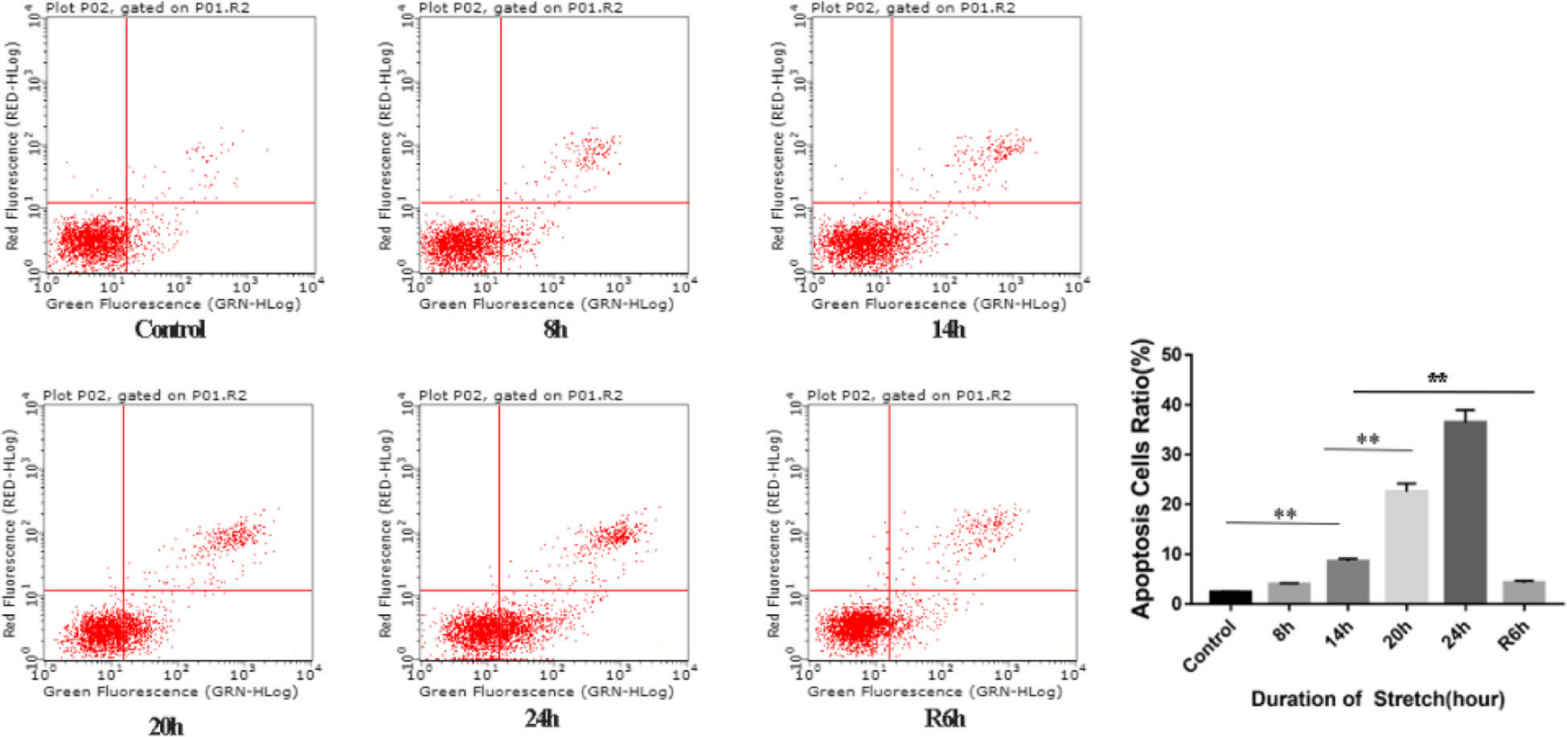
Stretch-induced apoptosis of RPE cells. Annexin V-PI double staining was used to detect the apoptosis of RPE cells at different time points. The apoptosis ratio was significantly increased at 14 h (*p* < 0.01) and reached its highest, 36.47%, at 24 h. In the stretch-withdrawn group, the apoptosis ratio significantly reduced at R6h compared to 14 h (*p* < 0.01). At R6h, the apoptosis ratio decreased to as low as 3.88%

**Fig. 4:**
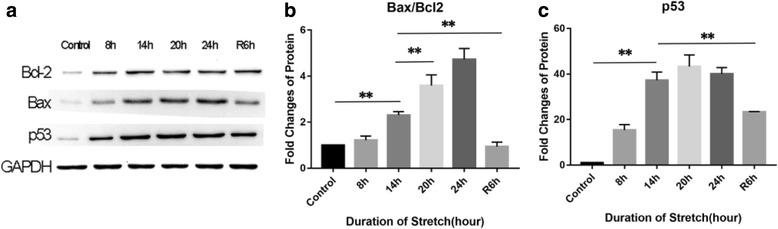
Western blotting analysis of apoptosis regulatory protein expression. **a** Coloured bands of Bcl2, Bax, and p53. **b**, **c** Expression levels of Bax, Bcl2 and p53. At 8 h, the Bcl2 and p53 expressions were significantly up-regulated (*p* < 0.01); the changes in Bax expression (*p* = 0.067) and the Bax/Bcl2 ratio (*p* = 0.957) were not statistically significant compared with the control group; however, thereafter, the Bax/Bcl2 ratio gradually increased with time. The expression of the p53 gene was significantly up-regulated at 14 h (*p* < 0.01). After stretch withdrawal, the Bax/Bcl2 ratio at R6h was significantly lower than that in another group where the stretch was not withdrawn (20 h) (*p* < 0.01), and the p53 expression was also significantly lower than in the stretch group (20 h) (*p* < 0.01)

Apoptosis can be induced by mechanical stress, so we performed Annexin V-PI double staining to detect the stretch-induced apoptosis of RPE cells. At 8 h of stretch, a slight increase was observed in the apoptosis ratio of RPE cells, but this change was not statistically significant compared with the control group. At 14 h, the stretch caused apparent apoptosis of RPE cells and the apoptosis ratio tended to increase with time (*p* < 0.01). In the stretch-withdrawn group, the apoptosis ratio significantly reduced at R6h (*p* < 0.01) compared with that at 14 h and 20 h (Fig. 3). In addition, the expression levels of apoptosis regulatory proteins Bcl-2 and Bax were also assessed. At 8 h, there was a slight increase in the expression of Bax, but this difference was not statistically significant compared with the control group (*p* = 0.067), whereas the expression of Bcl2 was significantly up-regulated (*p* < 0.01) (Fig. 4a). Meanwhile, the Bax/Bcl2 ratio exhibited an insignificant increase at 8 h (*p* = 0.957) and then continued to increase until 24 h (Fig. 4b). After withdrawal of the stretch, the Bax expression was down-regulated (Fig. 4a) and the Bax/Bcl2 ratio at R6h was significantly decreased compared to 14 h and 20 h (*p* < 0.01) (Fig. 4b). Additionally, the expression of the apoptosis-related gene p53 was significantly up-regulated at 14 h (*p* < 0.01) and remained relatively stable thereafter. After stretch withdrawal (R6h), the p53 expression was also significantly lower than at 14 h and 20 h (Fig. 4c).

### Changes in cytokine expressions

**Fig. 5:**
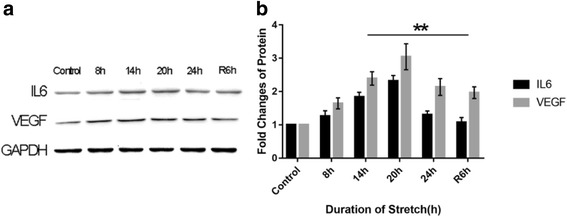
Western blotting analysis of IL6 and VEGF expression. **a** Colored bands of IL6 and VEGF; **b** Expression levels of IL6 and VEGF. The expression levels of both IL6 and VEGF increased with the duration of the stretch, reaching their peak levels at 20 h but showed significant reduction at 24 h (*p* < 0.01). At 6 h after withdrawal of the stretch (R6h), the IL6 and VEGF expressions were significantly down-regulated compared to 14 h (*p* < 0.01)

Under cyclic stretch, the IL6 expression in RPE cells was significantly up-regulated at 14 h compared with the control group (*p* < 0.01), and reached its peak at 20 h. After withdrawal of the stretch, the IL6 expression gradually returned to its normal level (Fig. 5b). The changes in the expression of VEGF were similar to those observed for IL6 (Fig. 5b). It should also be noted that the expression levels of both IL6 and VEGF at 24 h were significantly lower than those at 20 h (*p* < 0.01) (Fig. 5b).

## Discussion

In this study, RPE cells under cyclic stretch underwent morphological changes from a spreading shape to a rounded shape, and the expressions of integrin-FAK-Rac/cdc42 signalling pathway-related proteins that functioned to regulate cytoskeletal remodeling were also altered. A gradual increase in the apoptosis ratio was also observed, as well as changes in apoptosis regulatory proteins Bcl2, Bax and p53. In addition, the stretch resulted in up-regulation of the expression levels of inflammatory factor IL6 and vascular proliferation-related VEGF. After withdrawal of the stretch, these biological changes in cytoskeletal remodeling, apoptosis and cytokine secretion were gradually restored.

### Stretch-induced changes in cell morphology and cytoskeletal pathways

The cytoskeleton is a key structure helping cells to maintain their shape, adhesion and physiological functions. It is known that integrin is one of the major mechano-transducers for cell-matrix interactions [21]. In response to external stimuli, the cytoskeletal rearrangement is regulated via the integrin-FAK-Rac pathway [22], which is associated with morphological and functional changes in cells. In this study, we observed that the proportion of spreading-shaped RPE cells decreased with an increasing duration of stretch (Fig. 1a-f; Table 1) - it fell to 30% at 20 h. Meanwhile, the expression levels of integrin β3, pFAK397 and pRac were also significantly down-regulated at 20 h (Fig. 2). These findings indicate that the stretch may regulate cytoskeletal remodelling and be involved in maintaining cell shape via the integrin-FAK-Rac pathway.

A prior study showed that mechanical stretching of cells cultured on silicone rubber sheets caused stress fibres to realign perpendicular to the stretch direction [23]. In our study, stress fibre reorientation was not noted during the early stage of stretch (from 0 h to 8 h) (Fig. 1a-b), which may be associated with the stretch parameters we used. Abhishek Tondon and colleagues [24] reported that, at low stretch frequencies, myosin 2 protein could offset the tension changes induced by the stretch, keeping the cells under a relatively stable tension level without cytoskeletal rearrangement. Considering that, in real-world clinical settings, VMA produces continuous - but not static - stretch on the retinal area as the eyeball moves, we chose a relatively low stretch frequency (0.125 Hz) to better simulate reality.

### Stretch-induced RPE cell apoptosis

It has been demonstrated that physiological stretch has a protective effect on cells [25], whereas pathological stretch may induce cell apoptosis [26]. Based on Annexin-V/PI double staining, we found that the apoptosis ratio increased with the duration of the stretch (Table 2). At 8 h of stretch, most RPE cells maintained their spreading shape (Fig. 1b), despite an insignificant increase in the apoptosis ratio compared with the control group (Fig. 3, Table 2). Additionally, at 8 h, the expression of the apoptosis inhibitor Bcl-2 was up-regulated, but the Bax expression and the Bax/Bcl-2 ratio remained almost unchanged (Fig. 4b). These findings reveal that the stretch-induced apoptotic effect has not played a predominant role at 8 h.

**Table 2:** Apoptosis ratio of RPE cells

Duration of the stretch (hour)	Apoptosis ratio (%)
Control	2.15 ± 0.24
8 h	4.05 ± 1.03
14 h	11.06 ± 2.31
20 h	22.52 ± 1.68
24 h	36.47 ± 2.50
R6h	3.88 ± 0.77

Mayr and colleagues [26, 27] reported that mechanical stretch could mediate cell apoptosis via the integrin β1-rac-p53 pathway. In the present study, high expressions of pRac and pFAK397 were observed at 14 h (Fig. 2c-d). The p53 expression level, as well as the Bax/Bcl2 ratio, were also significantly elevated at 14 h compared with 8 h (both *p* < 0.01) (Fig. 4b-c). We speculate that the integrin β1-Rac-p53 pathway may play a role in stretch-induced RPE cell apoptosis.

After continuous stretching for 20-24 h, the expression levels of integrin, pRac and pFAK397 were significantly down-regulated (Fig. 2b-d), while the Bax/Bcl2 ratio and the p53 expression remained at high levels (Fig. 4b-c). This result indicates that other apoptotic pathways may be activated after long-term stretch [28]. Taken together, the stretch results in a time-dependent increase in apoptotic RPE cells, with the integrin β1-Rac-p53 pathway playing a role during the early stage of stretch, while other apoptotic signaling pathways may be activated to mediate RPE cell apoptosis after a long-term stretch.

### Stretch-induced changes in cytokine expressions

It has been shown that general mechanical stretch induces secretion and expression of VEGF by VEGF-expressing cells, such as cardiac myocytes [29] and mesangial cells [30]. In our study, the stretch caused time-dependent VEGF up-regulation in RPE cells (Fig. 5b), which is consistent with the findings reported by Seko and coworkers [31]. Interestingly, the IL6 and VEGF expressions were significantly down-regulated at 24 h (Fig. 5b), possibly because, in the presence of massive apoptosis, most RPE cells could not express or secrete cytokines normally. After withdrawal of the stretch, the expression levels of these cytokines were significantly lower than those in another group where the stretch was not withdrawn (*p* < 0.05); this indicates that IL6 and VEGF expression is closely related with mechanical strain.

The mechanism of stretch-induced VEGF up-regulation in RPE cells remains unclear. It has been shown that, in myocardial cells, stretch induces VEGF up-regulation via the TGF-β pathway [29]. In human mesangial cells, stretch activates the PKC and PTK pathways to up-regulate the VEGF expression [30]. In RPE cells, oxidative stress mediates the expression of VEGF via the TNF-α pathway [32]. These pathways may also be involved in stretch-induced VEGF up-regulation in RPE cells, but more studies are required to fully understand the underlying mechanism.

### Physiological restoration after stretch withdrawal

After withdrawal of the stretch, we observed obvious changes in the cell’s morphology, apoptosis ratio, and cytokine expression. In a study of human bladder smooth muscle cells [33], transient stretch induced acute cytoskeletal changes which could be completely restored within a short period of time and without causing extensive activation of signalling pathways. In our study, 14h continuous stretching had already induced a series of biological changes in RPE cells and activation of diverse signalling pathways; however, after stretch withdrawal, the morphological structure and physiological activities of these cells could still be gradually restored. These findings reveal that despite the pathological changes induced by the stretch, RPE cells are able to self-repair if the strain is inhibited within a certain timeframe. It can thus be inferred that inhibiting VMA-induced retinal stretching, by means of medication or surgery, has the potential to protect against the development and progression of AMD, which offers new insights into the prevention and treatment of AMD.

The RPE layer is the outer blood-retinal barrier that protects the health and integrity of the retina and choroid, regulates the volume and chemical composition of the subretinal space and regulates nutrient transport between the retina and choroid. In the pathogenesis of AMD, several risk factors, such as cellular ageing [13] and oxidative stress [34], may lead to impaired structural integrity, immunoregulation disturbance [35] and apoptosis [15] of RPE cells (and the onset of the disease). In this study, continuous cyclic stretch induced morphological changes of RPE cells and an increase in the apoptosis ratio, as well as up-regulation of the expression levels of IL6 and VEGF. All these biological changes were gradually reversed after withdrawal of the stretch; therefore, we speculate that stretch may impair the physiological functions of RPE cells and thus contribute to the pathogenesis and progression of AMD. VMA-induced retinal stretching may be considered as a potential controllable factor in the prevention and treatment of AMD.

## Conclusion

Mechanical stretch can induce morphological changes of RPE cells, cell apoptosis, and up-regulation of IL6 and VEGF; these changes are reversed upon withdrawal of the stretch.

## Abbreviations

ANOVA: One-way analysis of variance
ARPE-19: A spontaneously arising RPE cell line derived in 1986 by Amy Aotaki-Keen from the normal eyes of a 19-year-old male who died from head trauma in a motor vehicle accident
DMEM-F12: Dulbecco’s Modified Eagle Medium/Ham’s Nutrient Mixture F12
FBS: Fetal Bovine Serum
